# Can We Reduce Ports While Preserving Outcomes? A Case Series of 3-Port Laparoscopic Right Hemicolectomy with Intracorporeal Anastomosis

**DOI:** 10.5152/tjg.2026.25749

**Published:** 2026-02-02

**Authors:** Muhammet Kadri Çolakoğlu, Erdal Birol Bostancı

**Affiliations:** Department of Gastrointestinal Surgery, Health Sciences University, Ankara Bilkent City Hospital, Ankara, Türkiye

**Keywords:** 3-port laparoscopy, 3-port right hemicolectomy, colorectal cancer, reduced-port laparoscopic surgery

## Abstract

**Background/Aims:**

Three-port laparoscopic right hemicolectomy has emerged as an intermediate minimally invasive technique aimed at reducing port number while maintaining operative safety, ergonomics, and oncological adequacy. Evidence regarding its feasibility and clinical outcomes remains limited.

**Materials and Methods:**

A retrospective analysis was conducted on 42 consecutive patients who underwent 3-port laparoscopic right hemicolectomy with intracorporeal anastomosis for right-sided colon cancer between February 2023 and October 2025. Demographic characteristics, perioperative variables, postoperative recovery, complications, and pathological outcomes were evaluated.

**Results:**

The mean age was 66.5 years and the mean body mass index was 24.0 kg/m^2^. The hemicolectomy procedure was successfully completed using 3 ports in all patients. The mean operative time was 140 minutes, and the estimated blood loss was minimal, with no intraoperative transfusions. Oral intake resumed on postoperative day 1, and bowel function returned on the second day. The mean hospital stay was 7 days. Overall postoperative morbidity was 14.2%, consisting exclusively of minor complications; no anastomotic leaks or reoperations occurred. The mean lymph node yield was 22, with only 2 patients having fewer than 12 retrieved nodes; both of these patients had high-grade dysplasia rather than invasive carcinoma. All proximal and distal resection margins were oncologically adequate and microscopically negative.

**Conclusion:**

Three-port laparoscopic right hemicolectomy with intracorporeal anastomosis is technically feasible, safe, and oncologically adequate. This approach enables a reduction in port number without increasing complications or compromising specimen quality. It may represent a practical alternative in centers aiming to optimize minimally invasive techniques while preserving operative efficiency.

**Main Points**

- Three-port laparoscopic right hemicolectomy with intracorporeal anastomosis can be performed safely and efficiently, maintaining stable exposure and ergonomics despite reduced port number.
- The 3-port setup supports effective instrument maneuverability and surgeon autonomy, helping minimize external assistance without compromising operative control.
- Perioperative outcomes—including operative time, morbidity profile, lymph node retrieval, and margin status—were comparable to conventional multiport surgery, with no leaks observed in this series.
- Conversion to additional ports was uncommon and primarily related to concomitant procedures, suggesting that the 3-port approach remains reliable for isolated right hemicolectomy.
- The technique appears most suitable for non-obese patients without locally advanced tumors, and broader validation will require multicenter studies including higher-complexity cases.

## Introduction

Laparoscopic right hemicolectomy has become a standard approach for right-sided colon cancers, offering the advantages of minimal invasiveness, faster recovery, and comparable oncological outcomes to open surgery.^1^^-^^3^ Conventional laparoscopic techniques generally employ 4-5 trocars to facilitate adequate retraction, dissection, and specimen extraction. However, recent efforts have aimed to further reduce the number of access ports in order to simplify the procedure, minimize instrument collisions, and enhance surgeon autonomy, particularly in centers with limited personnel support.

Reducing the number of trocars in surgery has been associated with potential benefits, including decreased parietal trauma and improved cosmesis, although objective evidence supporting these outcomes remains limited.^4^ These investigations into right colon cancer have also been established in the literature. From a technical standpoint, port reduction may raise concerns regarding adequate exposure and traction during dissection, especially in obese patients or those with bulky mesenteric fat. Therefore, demonstrating the feasibility and oncological safety of reduced-port approaches remains essential before they can be widely adopted. In practice, when performing 3-port surgery, a right-handed primary surgeon typically uses the left hand for traction and the right hand for dissection, requiring only a camera assistant and no additional operative personnel. This setup can be advantageous in centers with limited staff availability or inexperienced assistants, as it minimizes the need for continuous verbal guidance and enhances the surgeon’s autonomy, potentially improving workflow efficiency and reducing operative time. Three-port laparoscopic right hemicolectomy represents an intermediate step between conventional multiport and single-incision techniques. It aims to maintain ergonomic efficiency while reducing dependence on an assistant. The introduction of intracorporeal anastomosis has further enhanced the minimally invasive nature of this operation by eliminating the need for exteriorization and allowing smaller extraction sites.

The present study reports a consecutive series of patients who underwent 3-port laparoscopic right hemicolectomy with intracorporeal anastomosis. The primary objective was to evaluate the technical feasibility and safety of this approach, while the secondary aim was to assess whether reducing the number of trocars compromises operative or oncological outcomes.

## Materials and Methods

This retrospective study included consecutive patients who underwent 3-port laparoscopic right hemicolectomy for a histologically confirmed right-sided colon tumor between February 2023 and October 2025. All procedures were performed by a single experienced gastrointestinal surgeon at a tertiary referral center. Patients with preoperative imaging suggesting locally advanced tumors involving adjacent organs, or those anticipated to require combined resections, were not selected for the 3-port technique.

All patients underwent standard preoperative assessment, including colonoscopy, contrast-enhanced abdominal computed tomography, and routine laboratory investigations. Demographic data, perioperative outcomes, and pathological findings were prospectively recorded in a departmental database and retrospectively analyzed for this study. The study protocol was approved by the institutional ethics committee of Health Science University, Ankara Bilkent City Hospital (Approval no.: E2-25-13441; Date: November 12, 2025), and all patients provided written informed consent for surgery and data use.

### Surgical Setup

All procedures were performed under general anesthesia with the patient positioned supine and both arms tucked. After induction, the operating table was placed in a slight Trendelenburg position with a left tilt to facilitate gravitational displacement of the small bowel. The operating surgeon consistently stood on the patient’s left side, and the entire operation was conducted using a strict 3-port configuration, supported only by a single camera assistant on the right.

### Port Placement and Exposure

**Figure 1.**
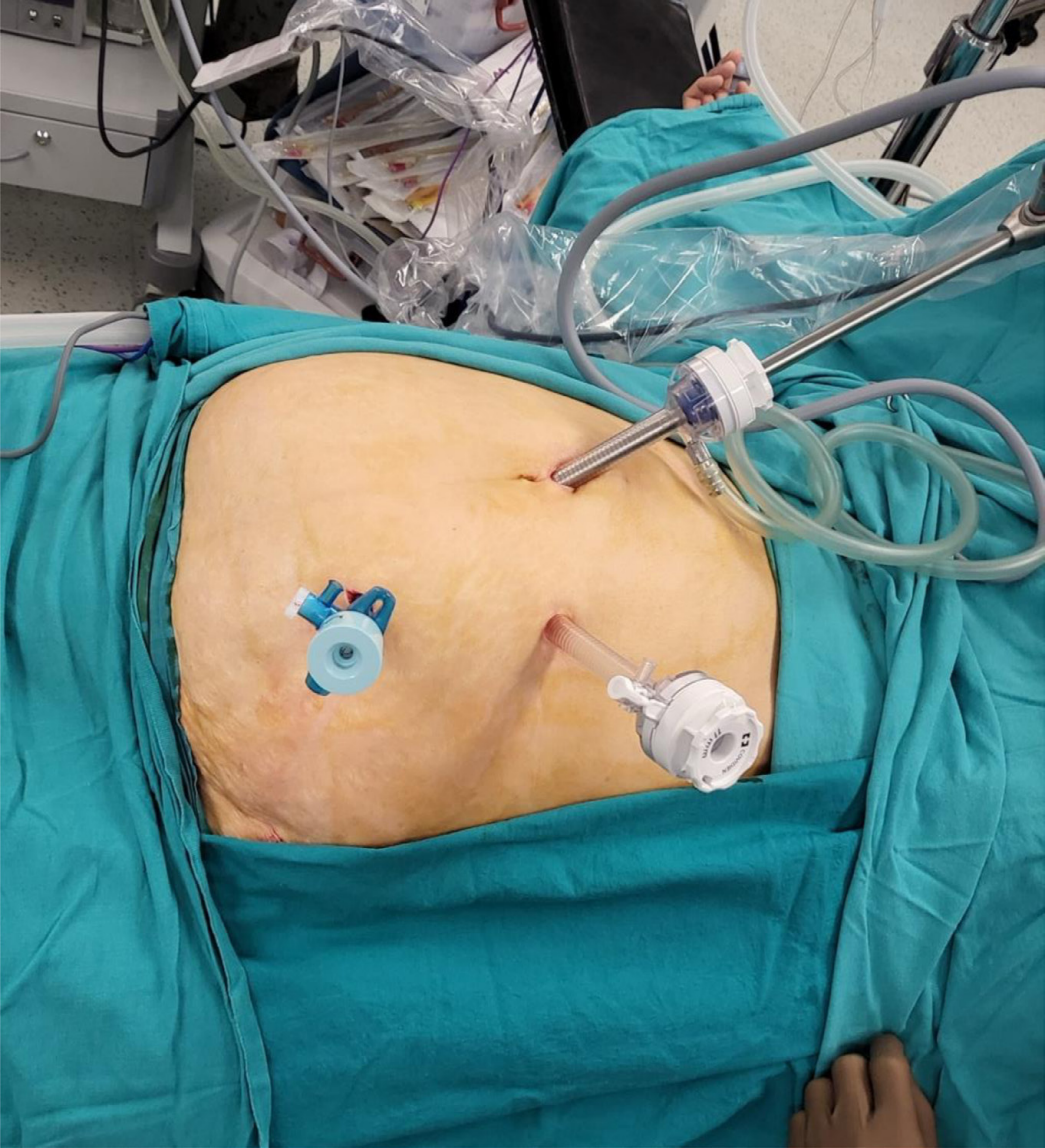
Port placement.

Pneumoperitoneum was established via a Veress needle technique. A 10-mm trocar for a 30° laparoscope was inserted just above the umbilicus and served as the central camera port. Two working trocars were placed in the left abdomen: a 5-mm trocar in the left lower quadrant and a 12-mm trocar in the left upper quadrant. This arrangement provided stable triangulation and allowed the surgeon to work ergonomically, using the left hand primarily for continuous traction and the right hand for precise dissection, without requiring additional ports or assistant-operated retractors (Figure 1).

Exploration of the peritoneal cavity was performed to rule out metastatic disease. The omentum and small bowel were swept toward the left upper quadrant to expose the ileocecal region and right colon. Importantly, no suspension sutures, clips, or extracorporeal traction devices were used to elevate the colon or mesentery; exposure was achieved solely through dynamic, surgeon-controlled traction via the 2 working instruments.

### Medial-to-Lateral Dissection

A standard medial-to-lateral approach was adopted. The peritoneum overlying the superior mesenteric vessels was incised to identify the ileocolic pedicle. Blunt and sharp dissection was performed strictly along the embryologic mesocolic plane, exposing the duodenum and the head of the pancreas. The ileocolic vessels were skeletonized and divided. When present, the right colic artery was similarly isolated and ligated according to oncological necessity. The dissection proceeded cranially toward the gastrocolic trunk. The right branch of the middle colic artery was identified and divided for an oncologically adequate resection. Throughout this phase, all traction was provided by the surgeon’s left hand, maintaining exposure without instrument crowding despite the reduced-port setup.

### Lateral Mobilization

After the medial mesocolic plane was developed and the posterior attachments were freed up to the hepatic flexure, the dissection continued laterally. The lateral peritoneal attachments (white line of Toldt) were divided from the cecum to the hepatic flexure. The omentum was separated from the transverse colon to complete mobilization. Full mobilization was achieved without the use of percutaneous sutures, towel clips, or abdominal wall suspension techniques, relying entirely on the surgeon’s controlled 2-instrument manipulation.

### Intracorporeal Anastomosis

Following complete mobilization, the terminal ileum and transverse colon were transected using linear staplers inserted through the 12-mm left upper quadrant port. An intracorporeal, isoperistaltic, side-to-side ileocolic anastomosis was fashioned with a linear stapler. The common enterotomy was closed with a running or interrupted absorbable suture.

### Specimen Extraction and Closure

The specimen was extracted through a 3-4 cm protected mini-incision, typically achieved by extending the umbilical trocar site. Fascia and skin were closed with standard techniques.

Throughout the entire operation, no auxiliary devices, articulating instruments, suspension sutures, or additional trocars were required. Exposure, retraction, and dissection were fully controlled by the surgeon through a pure 3-port technique, using left-hand traction and right-hand dissection.

### Statistical Analysis

Statistical analyses were performed using standard descriptive methods appropriate for retrospective observational data. Continuous variables—including age, body mass index (BMI), operative time, estimated blood loss, length of hospital stay, and lymph node count—were summarized as mean ± SD or median with range, depending on distribution. Categorical variables such as sex, American Society of Anesthesiologists (ASA) classification, tumor location, T- and N-stage, and postoperative complications were presented as frequencies and percentages.

Normality of distribution for continuous variables was assessed using the Shapiro–Wilk test and visual inspection of histograms. Because this study was designed as a descriptive case series without a comparison group, no inferential statistical testing (e.g., *t*-test, *χ*^2^ test) was performed. However, in secondary exploratory analysis, outcomes were reviewed qualitatively across BMI categories and tumor stages to identify potential patterns relevant to patient selection for 3-port surgery. All statistical calculations were performed using IBM SPSS Statistics for Mac version SPSS Statistics 24.0; (IBM SPSS Corp.; Armonk, NY, USA).

## Results

A total of 42 patients underwent 3-port laparoscopic right hemicolectomy with intracorporeal anastomosis. The median age was 66.5 years (range: 20-89), and the cohort consisted of an equal distribution of males and females (21 each, 50%). The median BMI was 24.0 kg/m^2^, with 71.4% of patients in the normal BMI range. The ASA scores were evenly distributed, with 50% classified as ASA I-II and 50% as ASA III-IV. Tumor location was the cecum in 40.6%, the ascending colon in 47.6%, and the hepatic flexure in 11.9% of patients (Table 1).

All procedures were initiated and completed using a standardized 3-port laparoscopic approach. Among the study population, 9 patients presented with concomitant cholelithiasis. In 3 of these cases, laparoscopic cholecystectomy was successfully performed through the same 3 ports without the need for additional trocars. In the remaining 6 patients, after completion of the right hemicolectomy and anastomosis, cholecystectomy was attempted, but technical difficulty—primarily due to limited retraction angles and adhesions—necessitated the placement of an additional 5-mm trocar to ensure safe dissection of Calot’s triangle. In one of these patients, despite the supplementary trocar, the gallbladder could not be safely dissected laparoscopically owing to dense inflammatory adhesions; therefore, the procedure was converted to an open cholecystectomy via a right subcostal incision.

The median operative time for right hemicolectomy and anastomosis was 140 minutes (range: 110-300). Estimated blood loss was low, with a median of 25 mL. Postoperative recovery was favorable: the median time to first flatus was 2 days, the median time to initiation of oral liquid intake was 1 day, and the median postoperative hospital stay was 7 days (range: 4-14). Postoperative complications occurred in 6 patients (14.2%), all graded as Clavien–Dindo I-II; no anastomotic leak, intra-abdominal abscess, or reoperation was observed. Complications included melena (n = 1), a postoperative C-Reactive Protein (CRP) elevation requiring antibiotic therapy (n = 1), chylous leak (n = 1), diarrhea (n = 1), anticoagulation adjustment (n = 1), and 1 wound-site infection (n = 1). All complications were managed successfully with medical and conservative treatment (Table 2).

Pathologic evaluation revealed adenocarcinoma in 78.5% of patients and high-grade dysplasia (HGD) in 21.4%. The median tumor size was 4 cm (range: 1-9.5). The median proximal and distal resection margins were 10.25 cm and 11.25 cm**,** respectively, and all margins were oncologically adequate and histologically negative. The median number of harvested lymph nodes was 22 (range: 7-74). Only 2 patients (5.2%) had fewer than 12 retrieved lymph nodes, and both had HGD rather than invasive carcinoma.

Regarding oncologic staging, 47.6% of patients had stage II disease, while stage I and stage III tumors accounted for 9.5% and 21.4% of cases, respectively; 21.4% were classified as stage 0 (high-grade dysplasia/Tis). T-stage distribution showed that 66.6% of tumors were T3-T4. Nodal staging demonstrated that 78.5% of patients were N0, while 16.6% were N1 and 4.7% were N2 (Table 3).

Overall, 3-port laparoscopic right hemicolectomy demonstrated favorable perioperative, pathological, and early oncologic outcomes, with complete oncologic resection achieved in all cases.

## Discussion

This study demonstrates that 3-port laparoscopic right hemicolectomy with intracorporeal anastomosis is a safe, feasible, and oncologically sound minimally invasive approach. Although reduced-port colorectal surgery has been explored for more than a decade, its integration with contemporary oncologic principles—particularly complete mesocolic excision (CME) and intracorporeal anastomosis—remains limited in the literature. The present series contributes to this field by showing that reducing the number of trocars does not compromise surgical exposure, mesocolic plane quality, lymph node harvest, or margin status.

One of the major concerns regarding reduced-port colectomy is the potential loss of triangulation, which is essential for safe vascular dissection and stable countertraction. Single-incision laparoscopic surgery, while cosmetically appealing, is often associated with longer operative times, increased instrument crowding, limited ergonomics, and a dependency on specialized articulating instruments. In contrast, the 3-port configuration used in this study preserves critical triangulation. The surgeon’s ability to perform left-hand traction and right-hand dissection**,** without the use of abdominal wall suspension sutures or accessory retraction devices, allows for a well-controlled operative field. Importantly, this approach enabled consistent identification of the ileocolic pedicle, the duodenum, and the pancreatic head within the correct embryological planes, demonstrating that CME principles can be reproduced reliably through 3 ports.

The findings of this study align with prior reports supporting the technical feasibility and oncological adequacy of 3-port colectomy. The pioneering work by Hasegawa et al^5^ in 2013 demonstrated that the 3-port technique is safe and reduces the need for manpower and equipment. In 2016, Wu et al^6^ compared 3-port and 4- to 5-port right hemicolectomies, showing that although some cases required additional ports or conversion, procedures completed with 3 ports were both feasible and oncologically safe. Further evidence from Kim et al,^7^ in a multicenter study comparing 74 3-port and 89 five-port cases, indicated that the 3-port technique may even offer advantages such as shorter operative time, higher lymph node yield, and reduced analgesic use. Consistent with this, Shi et al^8^ reported shorter operative time, reduced blood loss, and higher lymph node retrieval in the 3-port cohort in a propensity-matched analysis. Long-term oncologic data have also been encouraging; Zhang et al^9^ observed no differences in overall or disease-free survival between 3-port and five-port groups. Collectively, these studies support that port reduction does not inherently compromise oncologic quality.

Despite the increasing adoption of CME for right-sided colon cancer, reports of reduced-port CME remain sparse and are largely limited to isolated case videos. Bae et al^10^ (2020) and Ying et al^11^ (2021) each published video vignettes on 3-port CME with intracorporeal anastomosis, confirming feasibility in individual cases. More recently, Zhou et al^12^ (2023) presented a single-case reduced-port CME using a Pfannenstiel camera port, caudal approach, and percutaneous suspension sutures. In contrast, the present study’s technique demonstrates that right hemicolectomy can be performed without any such suspension sutures, extracorporeal traction devices, or auxiliary ports—achieving mesocolic integrity and central vascular control purely with surgeon-controlled 2-instrument manipulation.

Additionally, reduced-port techniques have been examined in combination with natural orifice specimen extraction. Efetov et al^13^ (2024) reported that reduced-port right colectomy with D3 lymphadenectomy and transvaginal specimen extraction resulted in shorter hospital stay, faster return of bowel function, and reduced early postoperative pain, without compromising lymph node yield or margin status. While this approach differs from ours, these data collectively highlight the expanding potential of reduced-port strategies within advanced minimally invasive colorectal surgery.

The low complication rate in the current series further supports the safety of the 3-port approach. Notably, no anastomotic leakage occurred—an outcome consistent with increasing evidence that intracorporeal anastomosis minimizes mesenteric traction, improves bowel alignment, and may reduce wound-related morbidity. Although postoperative pain and cosmetic outcomes were not formally evaluated, the reduction in port number and incision size suggests potential benefits that merit further prospective evaluation. Importantly, in 6 patients, an additional trocar was required to perform laparoscopic cholecystectomy unrelated to the colonic resection, and in 1 patient, a right subcostal incision was necessary due to severe inflammatory adhesions. These findings indicate that when planning a 3-port approach, concomitant pathologies and patient-specific anatomical factors must also be taken into account.

Despite these strengths, the study has limitations. Beyond the inherent constraints of its retrospective design and single-surgeon experience, the number of patients with high BMI and stage III disease was limited. Consequently, the findings cannot be generalized to all patient groups, particularly those with obesity or locally advanced tumors, in whom exposure and mesocolic traction may be more technically demanding. However, the data suggest that in non-obese patients and in tumors that are not locally advanced,the 3-port technique can be safely performed without compromising oncologic principles. Larger multicenter studies stratified by BMI, tumor stage, and anatomic complexity will be essential for refining patient selection.

In summary, this study supports 3-port laparoscopic right hemicolectomy with intracorporeal anastomosis as a practical, safe, and oncologically reliable alternative to conventional multiport colectomy. The technique preserves ergonomic efficiency and minimizes dependency on additional operative personnel. As minimally invasive colorectal surgery continues to evolve, the 3-port approach may represent an optimal balance between reduced access trauma and uncompromised surgical and oncologic rigor. This case series demonstrates that 3-port laparoscopic right hemicolectomy with intracorporeal anastomosis is a feasible, safe, and oncologically sound approach for selected patients with right-sided colon cancer. The technique preserves adequate triangulation and operative ergonomics, allowing reliable vascular control without the need for auxiliary ports, suspension sutures, or additional retraction devices. Operative time, postoperative recovery, morbidity, lymph node harvest, and margin status were all comparable to those reported in conventional multiport laparoscopic colectomy. While the results confirm that the 3-port approach can be performed without compromising oncologic quality, its applicability may not extend to all patients. The limited number of individuals with high BMI and stage III disease in this series precludes broad generalization. Nevertheless, the findings indicate that in non-obese patients and those without locally advanced tumors, 3-port right hemicolectomy can be safely and effectively performed.

## Figures and Tables

**Table 1.** Baseline Demographic and Clinical Characteristics of the Study Cohort

**Variable**	**Population** **(n = 42)**
Age (years) Median	66.5 (20-89)
Sex, n (%) Male(%) Female(%)	21 (50) 21 (50)
BMI, n (%) <19 19-25 >25	1 (2.3) 30 (71.4) 11 (26.1)
ASA score, n (%) I, II III, IV	21 (50) 21 (50)
Tumor site, n (%) Cecum Ascending colon Hepatic flexure	17 (40.6) 20 (47.6) 5 (11.9)

ASA, American Society of Anesthesiologists.

**Table 2.** Intraoperative and Early Postoperative Outcomes

**Variable**	**Population** **(n = 42)**
Operation time (min) Median	140 (110-300)
Estimated blood loss (mL) Median	25
Postoperative hospital stays (days) Median	7 (4-14)
Time to first flatus (days) Median	2
Time to liquid diet (days) Median	1
Complications, n (%)	6 (14.2)

**Table 3.** Pathologic and Oncologic Findings

**Variable**	**Population** **(n = 42)**
Tumor pathology, n (%) Adenocarcinoma High-grade dysplasia	33 (78.5) 9 (21.4)
Tumor size (cm) Median	4 (1-9.5)
Proximal resection margins (cm) Median	10.25
Distal resection margins (cm) Median	11.25
Number of lymph nodes Median	22 (7-74)
Number of metastatic lymph nodes Median	0 (0-7)
Tumor-Node-Metastasis stage, n (%) 0 I II III	9 (21.4) 4 (9.5) 20 (47.6) 9 (21.4)
T stage, n (%) is,1,2 3,4	14 (33.3) 28 (66.6)
N stage, n (%) 0 1 2	33 (78.5) 7 (16.6) 2 (4.7)

## Data Availability

The data that support the findings of this study are available on request from the corresponding author.
